# α-Ketoglutarate prevents hyperlipidemia-induced fatty liver mitochondrial dysfunction and oxidative stress by activating the AMPK-pgc-1α/Nrf2 pathway

**DOI:** 10.1016/j.redox.2024.103230

**Published:** 2024-06-13

**Authors:** Danyu Cheng, Mo Zhang, Yezi Zheng, Min Wang, Yilin Gao, Xudong Wang, Xuyun Liu, Weiqiang Lv, Xin Zeng, Konstantin N. Belosludtsev, Jiacan Su, Lin Zhao, Jiankang Liu

**Affiliations:** a Center for Mitochondrial Biology and Medicine, The Key Laboratory of Biomedical Information Engineering of Ministry of Education, School of Life Science and Technology, and Cardiometabolic Innovation Center of Ministry of Education, Department of Cardiology, and Department of Dermatology, First Affiliated Hospital, Xi'an Jiaotong University, Xi'an, Shaanxi, 710049, China; b Medical Research Center, Xi'an No.3 Hospital, The Affiliated Hospital of Northwest University, Xi'an, Shaanxi, 710018, China; c Department of Biochemistry, Cell Biology and Microbiology, Mari State University, Pl. Lenina 1, Yoshkar-Ola, 424001, Russia; d Institute of Theoretical and Experimental Biophysics, Russian Academy of Sciences, Institutskaya 3, Pushchino, 142290, Russia; e Department of Orthopaedics, Xinhua Hospital Affiliated to Shanghai Jiaotong University School of Medicine, Shanghai, 200092, China; f School of Health and Life Sciences, University of Health and Rehabilitation Sciences, Qingdao, Shandong, 266071, China

**Keywords:** α-Ketoglutarate (AKG), Hyperlipidemia, Fatty liver, Mitochondrial dysfunction, Oxidative stress, PGC-1α, Nrf2

## Abstract

α-Ketoglutarate (AKG), a crucial intermediate in the tricarboxylic acid cycle, has been demonstrated to mitigate hyperlipidemia-induced dyslipidemia and endothelial damage. While hyperlipidemia stands as a major trigger for non-alcoholic fatty liver disease, the protection of AKG on hyperlipidemia-induced hepatic metabolic disorders remains underexplored. This study aims to investigate the potential protective effects and mechanisms of AKG against hepatic lipid metabolic disorders caused by acute hyperlipidemia. Our observations indicate that AKG effectively alleviates hepatic lipid accumulation, mitochondrial dysfunction, and loss of redox homeostasis in P407-induced hyperlipidemia mice, as well as in palmitate-injured HepG2 cells and primary hepatocytes. Mechanistic insights reveal that the preventive effects are mediated by activating the AMPK-PGC-1α/Nrf2 pathway. In conclusion, our findings shed light on the role and mechanism of AKG in ameliorating abnormal lipid metabolic disorders in hyperlipidemia-induced fatty liver, suggesting that AKG, an endogenous mitochondrial nutrient, holds promising potential for addressing hyperlipidemia-induced fatty liver conditions.

## Highlights

• AKG prevents hyperlipidemia-induced hepatic lipid metabolic disorders in mice through the AMPK-PGC-1α/Nrf2 pathway.

## Graphical abstract

AKG regulates mitochondrial function and redox balance via activating the AMPK-PGC-1α/Nrf2 pathway to prevent hyperlipidemia-induced fatty liver.

**Figure:**
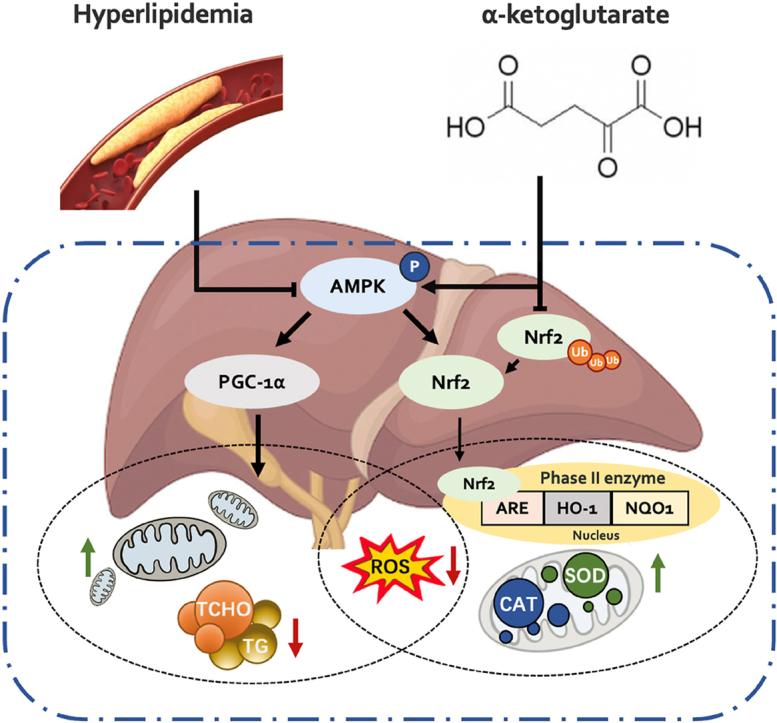

## 1 Introduction

Hyperlipidemia, a lipid metabolic disorder, is clinically characterized by an excess accumulation of total cholesterol, triglyceride, and low-density lipoprotein or a deficiency of high-density lipoprotein in serum [1]. Numerous studies have confirmed its prevalence across diverse populations, establishing it as the foundation for the development of various chronic diseases, including fatty liver, cardiovascular disease, and diabetes mellitus [2,3].

The correlation between hyperlipidemia and fatty liver infiltration is evident in hyperlipidemic patients. In the plasma of individuals with hyperlipidemia, increased triglyceride (TG) and cholesterol (T-CHO) levels disrupt the dynamic balance of hepatic fat and augment the production of non-esterified fatty acids (FFA). FFA, in turn, interferes with insulin-receptor binding, leading to insulin resistance and fat deposition in the liver. This, in consequence, disrupts intracellular membrane structures, including mitochondrial swelling, and causes the formation of reactive oxidative species (ROS), cytokine production, and damage to hepatocytes [4]. Nonionic surfactant Poloxamer 407 (P407), a hyperlipidemia model inducer, could impair organs, including the liver [5,6]. It is deemed to induce rapid lipid elevation in a short period (24 h), but the profile gradually normalized with increased treatments (10 d) [7,8]. Previous studies conducted by our laboratory indicated that a 24 h intraperitoneal injection of P407 induces lipid elevation, accompanied by hepatic fatty infiltration, presenting features akin to non-alcoholic fatty liver disease (NAFLD). Additionally, this induction of lipid elevation is associated with hepatic oxidative stress and mitochondrial dysfunction [6,9].

α-Ketoglutarate (AKG), a key intermediate in the tricarboxylic acid (TCA) cycle, connects intracellular carbon-nitrogen metabolism. It positively affects various tissues, including the heart, liver, kidney, and so on [10]. In the P407-induced hyperlipidemia mouse model, AKG ameliorated endothelial damage and inflammation by inhibiting mitochondrial dysfunction and oxidative stress [11]. Furthermore, circulation AKG levels were negatively correlated with blood glucose in diet-induced obese mice, while supplementation of water containing 2 % AKG significantly increased plasma AKG levels and decreased liver gluconeogenesis [12]. However, the effects of AKG on hyperlipidemia-induced hepatic lipid metabolic disorders have not yet been studied.

Mitochondria, serving as critical organelles in cells for energy production, also function as stress sensors and effectors. Depletion of mitochondrial number or function in cells leads to the overproduction of ROS, subsequently inducing chronic diseases such as cardiovascular disease, NAFLD, and diabetes [[13], [14], [15], [16]]. The peroxisome proliferator-activated receptor γ-coactivator 1-α (PGC-1α) is a major player in mitochondrial biogenesis regulation. In NAFLD patients, liver-specific PGC-1α deficiency leads to impaired hepatic mitochondrial oxidative capacity and hepatic steatosis [17]. It has been reported that activated PGC-1α by AMP-activated protein kinase (AMPK) phosphorylation reduces lipid accumulation and improves mitochondrial function, as well as enhances the activities of antioxidant proteins such as Sirt3 and NF-E2-related factor 2 (Nrf2) [18]. It is argued that Nrf2 plays a role in mitochondrial biogenesis and may form a feedback loop with PGC-1α [19,20]. Although the activation of the AMPK-PGC-1α/Nrf2 pathway has emerged as a promising strategy for treating metabolic disorder-related diseases, including NAFLD, the effect of AKG in this pathway remains unknown.

As a key transcription factor in antioxidant stress regulation, Nrf2 is degraded primarily through the ubiquitination-mediated proteasomal pathway, a process tightly regulated by E3 ubiquitin ligase Keap1. After uncoupling with Keap1, Nrf2 is activated and transported into the nucleus to initiate the expression of downstream antioxidant enzymes. Recent studies reported that deletion of Ubiquitin-specific peptidase 25 against acute liver injury induced by acetaminophen, reduces Nrf2 ubiquitination levels and increases Nrf2 content, enabling hepatocytes to better cope with oxidative damage [21]. Indeed, Nrf2 activators are valuable in modulating lipid metabolism and oxidative stress in hepatocytes to alleviate fatty liver in mice [22], whereas the role of AKG in the regulation of liver Nrf2 is unclear.

In this study, we propose that AKG may prevent the abnormalities in hepatic lipid metabolism induced by hyperlipidemia, along with its associated mitochondrial dysfunction and oxidative stress. This potential effect is proven to occur through activating the AMPK-PGC-1α/Nrf2 pathway.

## 2 Results

### 2.1 AKG pretreatment attenuates hepatic lipid metabolic disorders in P407-induced hyperlipidemia mice

**Fig. 1:**
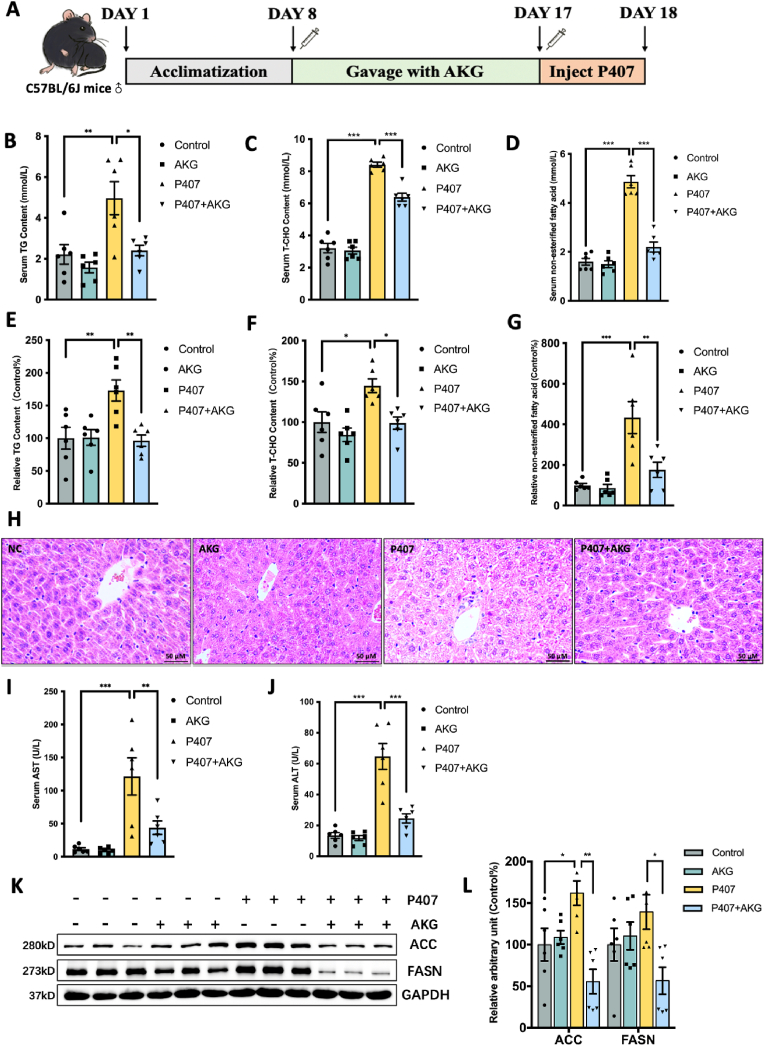
**AKG pretreatment attenuates hepatic lipid metabolic disorders in P407-induced hyperlipidemia mice**. (A) Schematic representation of animal experiments. Male C57BL/6J mice (8 weeks old) were administered with either ddH_2_O or AKG (50 mg/kg/day) for 9 days, followed by intraperitoneal injection with 0.5 g/kg P407 for 24 h. Serum (B) TG content, (C) T-CHO content, (D) FFA content, Relative liver (E) TG content, (F) T-CHO content, (G) FFA content, (H) H&E staining of liver tissue, Serum (I) AST content, (J) ALT content, (K) Western blot image and (L) relative protein content statistical analysis of ACC and FASN. The values are means ± SEM, n = 6. *p < 0.05, **p < 0.01, and ***p < 0.001.

Intraperitoneal injection of P407 for 24 h induces hyperlipidemia in mice (Fig. 1A), as evidenced by elevated plasma triglycerides and cholesterol levels [11]. Pretreatment with 50 mg/kg/day AKG not only significantly inhibited serum lipid increases but also mitigated the elevation of TG (Fig. 1B–E), T-CHO (Fig. 1C–F), and FFA levels in the liver (Fig. 1D–G). H&E staining revealed that P407-treated mice exhibited hepatocyte steatosis, with lipids accumulating in cells to form vacuoles. However, AKG pretreatment prevented the injury caused by P407, reducing the number of interstitial vacuoles (Fig. 1H). Moreover, P407 injection increased plasma AST and ALT levels, and AKG pretreatment ameliorated prevented the increases (Fig. 1I and J). Additionally, two key regulators of fatty acid synthesis, ACC and FASN, were upregulated in the P407 group but showed recovery in the AKG-pretreated group (Fig. 1K and L).

### 2.2 AKG pretreatment improves hepatic mitochondrial dysfunction in P407-induced hyperlipidemia mice

**Fig. 2:**
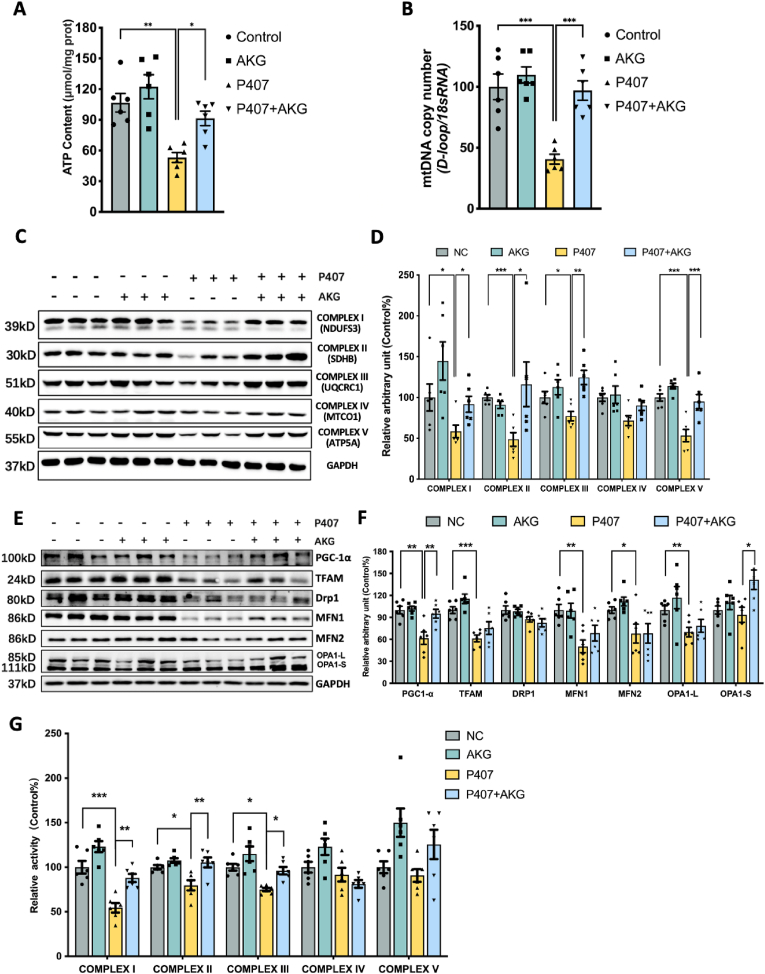
**AKG pretreatment improves hepatic mitochondrial dysfunction in P407-induced hyperlipidemia mice.** Liver (A) ATP content, (B) Relative mtDNA copy number, (C) Western blot image and (D) statistical analysis on relative protein content of complexes I–V in mouse liver, (E) Western blot image and (F) statistical analysis on relative protein content of PGC-1α, TFAM, Drp1, MFN1, MFN2, OPA1-L, OPA1-S in mouse liver, (G) Relative activity of mitochondrial complex I–V in hyperlipidemic mouse liver. The values are means ± SEM,c n = 6. *p < 0.05, **p < 0.01, and ***p < 0.001.

Fatty accumulation has been identified as a contributor to mitochondrial dysfunction [23]. In this study, we investigated the impact of AKG on liver mitochondrial function. As illustrated in Fig. 2A and B, the ATP content and mtDNA copy number, suppressed by P407, were effectively protected by AKG pretreatment. On the protein level, P407 injection significantly reduced the levels of mitochondrial subunits of complex I (NDUFS3), II (SDHB), III (MTCO1), and V (ATP5A). However, pretreatment with 50 mg/kg AKG restored these mitochondrial complex subunits (Fig. 2C and D). Remarkably, P407 had a substantial impact on the mitochondrial biogenesis factor PGC-1α and its downstream proteins, including TFAM, MFN1, and MFN2, as well as the mitochondria fusion factor OPA1. AKG pretreatment played a significant role in recovering the protein levels of PGC-1α and the short isoform of OPA1 (OPA1-S), slightly rescuing the decline of TFAM, MFN1, and the long isoform of OPA1 (OPA1-L) (Fig. 2E and F). Recognizing the crucial role of mitochondrial complex activities in mitochondrial function, we further measured the effect of AKG on complex activity. The results revealed that compared to the P407 group, AKG pretreatment significantly alleviated the downtrend activity of complexes I, II, III, and slightly improved V (Fig. 2G).

### 2.3 AKG pretreatment inhibits the loss of hepatic redox homeostasis in P407-induced hyperlipidemia mice

**Fig. 3:**
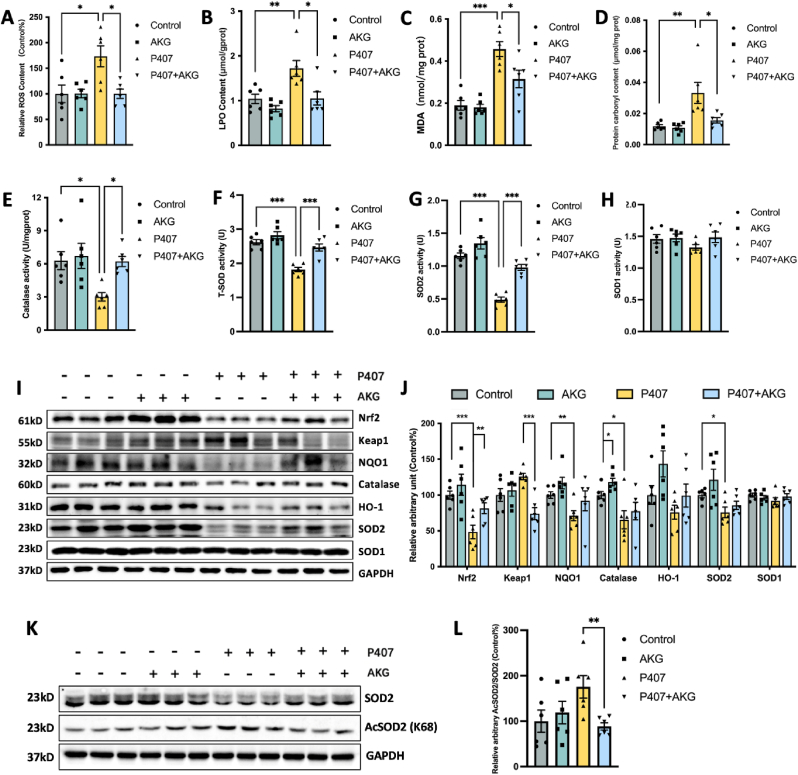
**AKG pretreatment inhibits the loss of hepatic redox homeostasis in P407-induced hyperlipidemia mice**. Liver (A) ROS, (B) LPO, (C) MDA, (D) protein carbonyl, (E) catalase activity, (F) total SOD activity, (G) SOD2 activity (H) and SOD1 activity. (I) Western blot image and (J) Statistical analysis on the relative protein content of Nrf2, Keap1, NQO1, Catalase, HO-1, SOD2, and SOD1 in the hyperlipidemic mouse liver. (K) Western blot image of SOD2 and AcSOD2, (L) statistical analysis on the relative protein content of AcSOD2/SOD2 in the liver. The values are means ± SEM, n = 6. *p < 0.05, **p < 0.01, and ***p < 0.001.

Mitochondrial impact on redox biomarkers in steatohepatitis [24] prompted us to assess the influence of AKG on these markers in the livers of hyperlipidemia mice. Compared to untreated mice, the P407 treatment significantly increased ROS, LPO, MDA, and protein carbonyl levels, while AKG pretreatment greatly alleviated their accumulation (Fig. 3A–D). Concurrently, P407 inhibited the activities of the antioxidant enzymes Catalase and total SOD while AKG pretreatment prevented the P407-induced decrease in activities of Catalase and total SOD (Fig. 3E and F). SOD mainly contains Cu-SOD (SOD1) and Mn-SOD (SOD2). Interestingly, AKG significantly restored hepatic SOD2 activity in hyperlipidemic mice but had no significant effect on SOD1 activity (Fig. 3G and H). Further examination of AKG effects on the vital antioxidant regulator Nrf2 and its mediated phase II system proteins in liver tissue revealed that AKG pretreatment significantly decreased the protein content of Keap1 but increased the Nrf2 in P407-treated mice, also caused trends of increase in the protein levels of antioxidant enzymes NQO1, catalase, and SOD2 (Fig. 3I and J). Furthermore, we noted that although AKG supplementation promoted the up-regulation of SOD2 protein expression, the amplification of its activity was even more remarkable, implying that post-translational modification of SOD2 may have occurred. Thus, we examined the alteration of SOD2 acetylation after AKG treatment, which showed that AKG inhibited the acetylation of SOD2. All these findings suggest that AKG can regulate hepatic redox homeostasis in hyperlipidemia.

### 2.4 AKG pretreatment attenuates PA-induced lipid metabolic disorders, mitochondrial dysfunction, and oxidative stress in HepG2 cells and primary hepatocytes

**Fig. 4:**
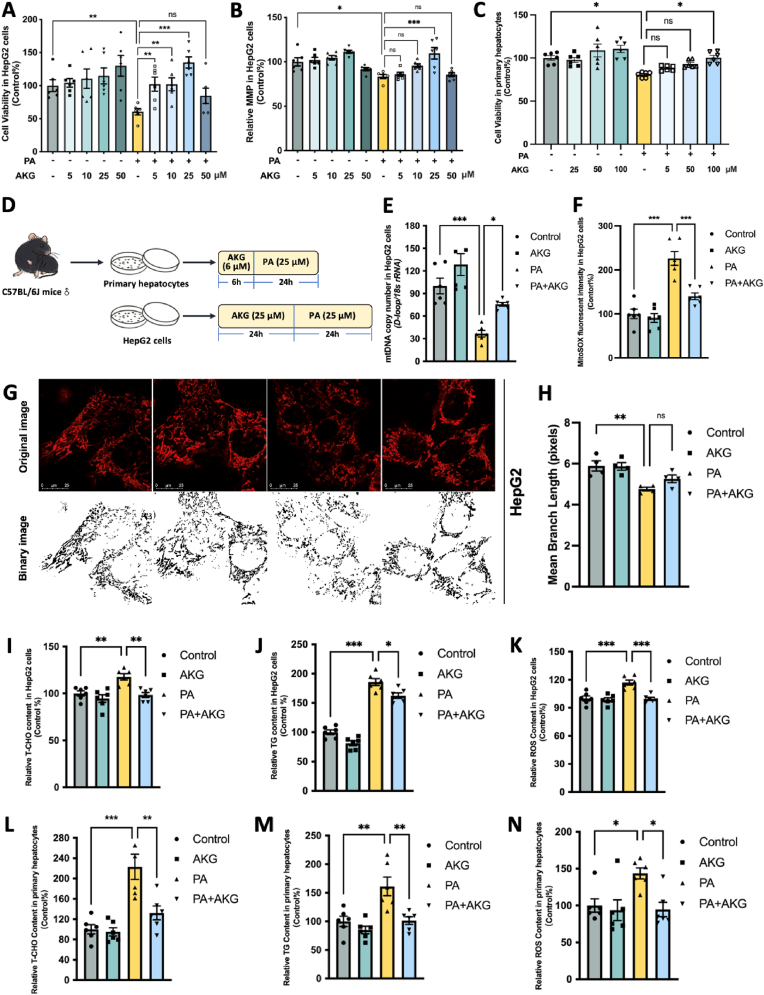
**AKG pretreatment attenuates PA-induced lipid metabolic disorders, mitochondrial dysfunction, and oxidative stress in HepG2 cells and primary hepatocytes.** HepG2 cells were pretreated with AKG (5, 10, 25, and 50 μM) or AKG (25 μM) for 24 h, followed by PA (250 μM) challenge for another 24 h. (A) Cell viability and (B) MMP in HepG2 cells. Primary hepatocytes were pretreated with AKG (25, 50,100 μM) for 6 h, followed by PA (250 μM) challenge for another 24 h. (C) Cell viability in Primary hepatocytes. (D) Schematic representation of follow-up cell experiments. (E) mtDNA copy number, and (F) Relative mitochondria ROS determined by MitoSOX in HepG2 cells. (G) Mitochondrial morphology (stained by Mito-tracker Red) and (H) mean branch length in HepG2 cells. Relative (I) T-CHO, (J) TG, (K) ROS in HepG2 cells. Relative (L) T-CHO, (M) TG, (N) ROS in primary hepatocytes. The values are means ± SEM from at least three independent experiments. *p < 0.05, **p < 0.01, and ***p < 0.001. (For interpretation of the references to colour in this figure legend, the reader is referred to the Web version of this article.)

To delve into the specific molecular mechanisms through which AKG alleviates hepatic lipid metabolic disorders, we conducted studies using HepG2 cells and primary liver cells. Based on the results of MTT and MMP assays, 24 h AKG treatment significantly preserved cell viability and MMP in a dose-dependent manner following PA injury in HepG2 cells, with the optimum protection observed at a concentration of 25 μM (Fig. 4A–B and D). In the meanwhile, 6 h AKG pretreatment could protect PA-injured primary hepatocytes cell viability in a dose-dependent manner and the protective effect of 100 μM AKG pretreatment is markedly, which used as optimal dose in follow up primary liver cell experiments (Fig. 4C and D). Next, we found that the AKG (25 μM) pretreatment prevented the PA-induced decrease in mtDNA copy number (Fig. 4E) but suppressed the increase in MitoSOX fluorescence intensity (Fig. 4F) in HepG2 cells. Observations of mitochondrial dye Mito-tracker Red staining revealed a fragmented morphology in the mitochondria of HepG2 cells after PA treatment. However, pre-treatment with AKG significantly improved the integrity of mitochondrial morphology (Fig. 4G and H). Moreover, AKG ameliorated PA-induced increases in T-CHO (Fig. 4I), TG (Fig. 4J), as well as ROS (Fig. 4K) in HepG2 cells. Similar results were observed in lipid overload primary hepatocytes that pretreated with 6 h of 100 μM AKG suppressed the accumulation of T-CHO (Fig. 4L), TG (Fig. 4M), and ROS (Fig. 4N). These findings suggest that AKG *in vitro* effectively mitigates lipid accumulation, oxidative stress, and mitochondira function in hepatocytes.

### 2.5 AKG pretreatment attenuates PA-induced hepatocyte dysfunction through PGC-1α and Nrf2 system in HepG2 cells and primary hepatocytes

**Fig. 5:**
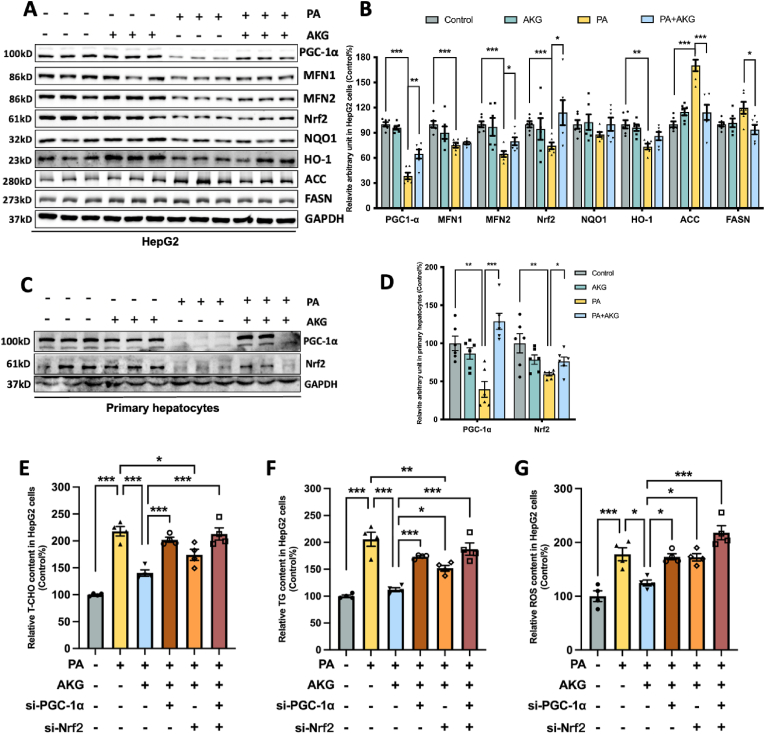
**AKG pretreatment attenuates PA-induced hepatocyte dysfunction through PGC-1α.** (A) Western blot image and (B) Statistical analysis on relative protein levels of PGC-1α, MFN1, MFN2, Nrf2, NQO1, HO-1, ACC, and FASN in the HepG2 cells. (C) Western blot image and (D) Statistical analysis on relative protein levels of PGC-1α and Nrf2 in primary hepatocytes. HepG2 cells were treated with the Ctrl siRNA, PGC-1α siRNA, or/and Nrf2 siRNA before adding AKG and PA for 48 h. (E) Relative T-CHO, (F) Relative TG, (G) Relative ROS content. The values are means ± SEM from at least three independent experiments. *p < 0.05, **p < 0.01, and ***p < 0.001.

In HepG2 cells, PA significantly impaired the protein expression of PGC-1α, MFN1, MFN2, Nrf2, and HO-1, while markedly increasing ACC protein levels. However, AKG pretreatment effectively restored mitochondrial PGC-1α, MFN2, and Nrf2 protein levels, slightly restoring MFN1, NQO1, and HO-1 levels and significantly suppressing the expression of ACC and FASN (Fig. 5A and B). AKG pretreatment similarly prevented the reductions of PGC-1α and Nrf2 protein levels in primary liver cells (Fig. 5C and D). These findings are consistent with *in vivo* results. To understand the roles of PGC-1α and Nrf2, two core proteins influencing mitochondrial function and redox homeostasis in the protective effects of AKG, we knocked down their expression through siRNA transfection (Su-Fig. A-B) and then assessed the effects of AKG on T-CHO, TG and ROS. It was found that the suppressive effects of AKG on T-CHO, TG and ROS were significantly counteracted after PGC-1α was knocked down, while the mitigating effects of AKG on ROS and T-CHO accumulation were also suppressed after Nrf2 was knocked down (Fig. 5E–G). The effect on eliminating the protective effect of AKG was more pronounced after knockdown of both PGC-1α and Nrf2, indicating that both PGC-1α and Nrf2 are involved in the regulation of AKG's protective role on lipid overload hepatic.

### 2.6 AKG pretreatment inhibits the ubiquitination of endogenous Nrf2 in lipid-overloaded mouse liver

**Fig. 6:**
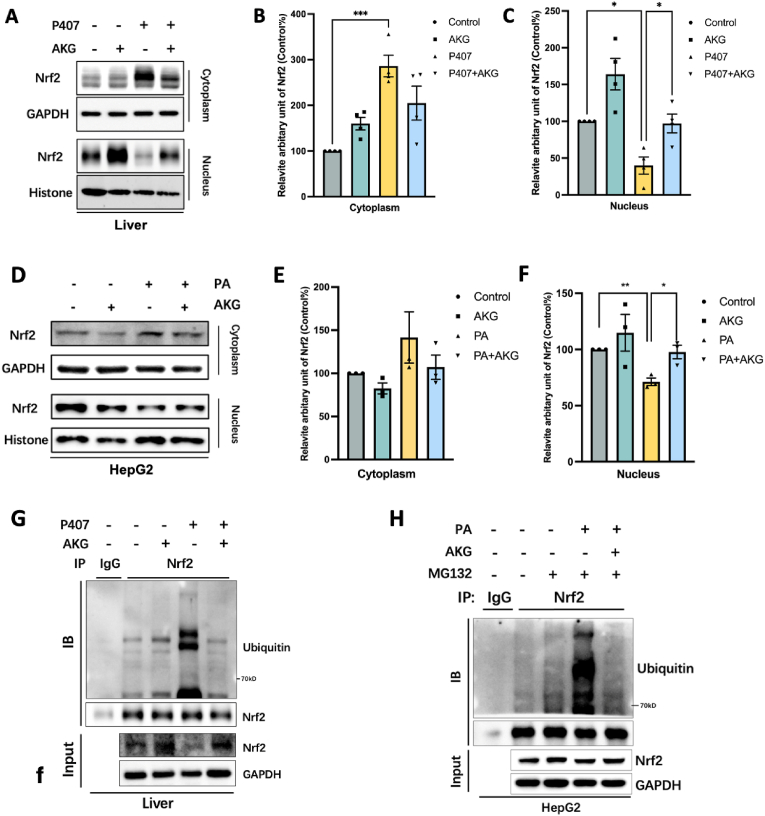
**AKG pretreatment inhibits the ubiquitination of endogenous Nrf2 in lipid-overloaded mouse liver.** (A) Western blot image and (B) Statistical analysis on the relative protein content of Nrf2 in the cytoplasm and nuclear in the liver. (C) Western blot image and (D) Statistical analysis on the relative protein content of Nrf2 in the cytoplasm and nuclear in the HepG2 cells. (E) Immunoprecipitated Nrf2 in liver tissue then detect the ubiquitination of endogenous Nrf2 by Immunoblot analysis. Add 20 μM MG132 to cells 6 h before collection. (F) Immunoprecipitated Nrf2 in HepG2 cells then detect the ubiquitination of endogenous Nrf2 by Immunoblot analysis. The values are means ± SEM from at least three independent experiments. *p < 0.05, **p < 0.01, and ***p < 0.001.

Earlier we found that AKG activated Nrf2 while decreasing the expression of Keap1, a protein that mediates the ubiquitination degradation of Nrf2 (Fig. 3I–G). We then assayed whether supplementation of AKG could alter the localization of Nrf2 within lipid-overloaded hepatocytes. It was found that both in lipid-overloaded mose liver tissues (Fig. 6A–C) and HepG2 cells (Fig. 6D–F), AKG pretreatment significantly promoted Nrf2 content in the nucleus. Next, the effect of AKG on the ubiquitination of endogenous Nrf2 in lipid-overloaded mouse liver and HepG2 cells was examined by immunoprecipitation experiments. As expected, both *in vivo* (Fig. 6G) and *in vitro* (Fig. 6H), we detected the effect of AKG on lipid overload-induced deubiquitination of hepatic Nrf2. Here, we also tested the effect of AKG on phosphorylation, another common post-translational modification, on two key molecules of our interest, PGC-1α and Nrf2. As shown in Supplementary Figure C and D,AKG had no significant effect on the phosphorylation of PGC-1α and Nrf2.

### 2.7 AKG pretreatment activates PGC-1α/Nrf2 by regulating AMPK phosphorylation

**Fig. 7:**
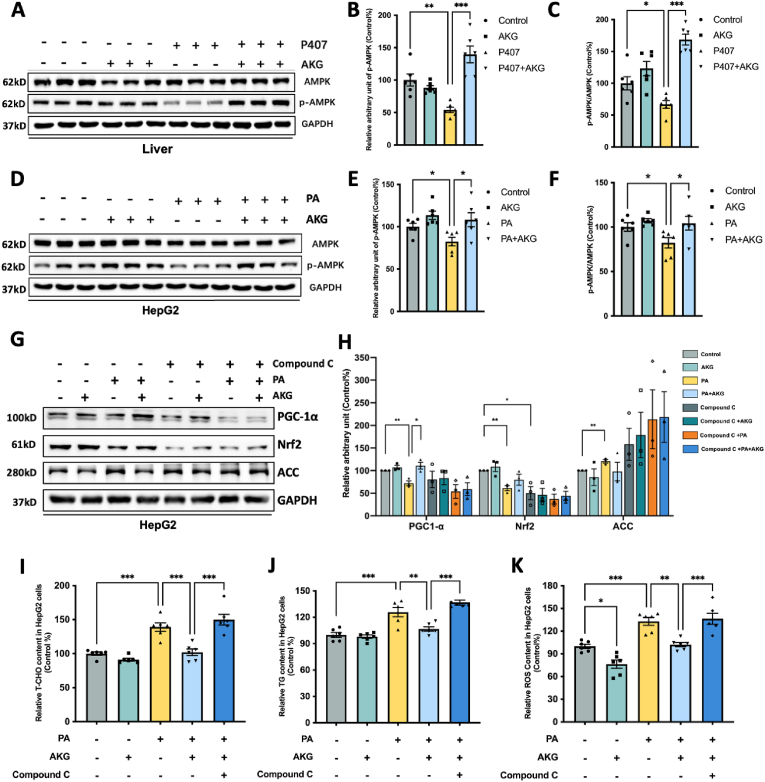
**AKG pretreatment activates PGC-1α/Nrf2 by regulating AMPK phosphorylation**. (A) Western blot image of AMPK and *p*-AMPK, statistical analysis on the relative protein content of (B) *p*-AMPK/AMPK and (C) *p*-AMPK in mouse liver, (D) Western blot image of AMPK and *p*-AMPK, statistical analysis on the relative protein levels of (E) *p*-AMPK/AMPK and (F) *p*-AMPK in HepG2 cells. HepG2 cells were treated with or without AMPK inhibitor Compound C (10 μM) before adding AKG (25 μM) for 1h. (G) Western blot image and (H) Statistical analysis on PGC-1α, Nrf2, ACC. (I) Relative T-CHO, (J) Relative TG, (K) Relative ROS in HepG2 cells that inhibited AMPK phosphorylation. The values are means ± SEM from at least three independent experiments. *p < 0.05, **p < 0.01, and ***p < 0.001.

Investigating the impact of AKG on AMPK activation in both metabolically abnormal livers *in vivo* and hepatocytes *in vitro*, we focused on the phosphorylation of AMPK, which promotes the expression of PGC-1α and Nrf2 [24]. In the mouse liver, AKG suppressed the AMPK phosphorylation inhibition induced by P407 (Fig. 7A–C), while no significant effect on AMPK itself (Su-Fig. E). Similar findings were observed in HepG2 cells, where AKG pretreatment restored the PA-induced decrease in *p*-AMPK protein level and the *p*-AMPK/AMPK ratio but AMPK is not changed (Fig. 7D–F and Su-Fig. F). To ascertain whether AMPK activation is the key factor in the liver protection of AKG, we used compound C to inhibit AMPK phosphorylation (Su-Fig. G-H). The results indicated that the suppressive effects of AKG on T-CHO (Fig. 7I), TG (Fig. 7J), and ROS (Fig. 7K) were significantly abolished after inhibiting AMPK activation. Meanwhile, we evaluated the effects of AMPK activation on PGC-1α and Nrf2. The results revealed that the activation of AKG on PGC-1α and Nrf2 was nullified after AMPK phosphorylation inhibition in PA-injured HepG2 cells. Additionally, the suppressive effect of AKG on ACC was counteracted by the inhibition of AMPK phosphorylation (Fig. 7G and H). These findings suggest that AKG ameliorates hepatic lipid metabolism abnormalities through the AMPK-PGC-1α/Nrf2 pathway.

## 3 Discussion

Hyperlipidemia, characterized by elevated blood lipid levels, is commonly associated with developing metabolic diseases such as fatty liver [25]. The molecular mechanisms linking hyperlipidemia to NAFLD remain unclear. The current study examined the impact of AKG, an essential intermediate product of the TCA cycle, on hyperlipidemia-induced fatty liver in mice. AKG prevented P407-induced hepatic lipid metabolic abnormalities in mice by modulating mitochondrial function and redox homeostasis through the activation of the AMPK-PGC-1α/Nrf2 pathway.

Intermediates of the TCA cycle, including AKG, have physiological functions such as regulating energy metabolism [26]. As a pivotal intermediate linking carbon and nitrogen metabolism, AKG plays a critical role in regulating fatty acid synthesis, glucose homeostasis, and amino acid metabolism. It has been reported to protect against a wide range of diseases such as cardiovascular, brain, and renal ailments [10]. Our recent study found that the plasma level of AKG was decreased in the P407-induced hyperlipidemic mice and 50 mg/kg daily dose of AKG reduced plasma lipid level, suggesting a link between AKG and lipid metabolism. Here, we found that AKG reduced lipid deposition in the fatty livers, simultaneously inhibited the marked increase in the activities of AST and ALT, the key enzymes used to assess liver function, also ameliorated hepatocyte steatosis around the central vein in mice. In fact, the therapeutic potential of AKG has been underscored by recent findings in diet-induced obese mice, where circulating AKG level was negatively correlated with blood glucose levels [12]. Dietary supplementation with AKG improved systemic insulin sensitivity by enhancing glucose and fatty acid oxidation in skeletal muscle [27]. It is a remarkable fact that up to 70 % of the type 2 diabetes patient population is comorbid with fatty liver, that adiposity AKG inhibition may be a secondary response to the hypoglycemia effect [28]. Further examination revealed that AKG inhibited the protein expressions of FASN and ACC, core regulators of fatty acid synthesis, deciphering the effect of AKG on hepatic lipid metabolism. These findings collectively underscore the promising role of AKG in alleviating disorders of hepatic lipid metabolism.

As the main site of fatty acid oxidation, the structural integrity and functional completeness of mitochondria are vital for the normal physiological function of hepatocytes, while mitochondrial structural injury and dysfunction may result in the pathologic manifestations of NAFLD [23,24]. We observed that either in the P407-induced mouse model of hyperlipidemia or PA-injured hepatocyte model, mtDNA copy number decrease was prevented by AKG pretreatment. Experiments at the protein level further supported this result, suggesting that AKG protected against the downtrend in expression of PGC-1α, a key protein for mitochondrial biosynthesis, and its downstream factor TFAM, a regulator of the replication and transcription of mtDNA. Additionally, AKG pretreatment effectively protected the integrity of mitochondrial morphology and promoted mitochondrial fusion, but had no significant effect on mitochondrial fission. These findings suggest the role of AKG in ameliorating P407-induced liver injury by restoring mitochondrial mass and number, particularly by enhancing mitochondrial biogenesis ability.

AKG is regarded as a supplement that improves muscle energy metabolism and enhances muscular strength by increasing ATP levels in muscle [29]. We proved that AKG supplementation significantly augments ATP content in the fatty liver. ATP synthesis depends on MMP, which is also protected by AKG in PA-injured HepG2 cells. It has been reported that upregulation of the TCA cycle by AKG induces the production of more reducing equivalents, which are further utilized by the mitochondrial electron transport chain (ETC) to generate ATP [30]. As expected, AKG pretreatment significantly prevented the impairment in the protein content of complex I (NDUFS3), II (SDHB), III (MTCO1), and V (ATP5A), also prevented the activity of complex I, II, and III, reconfirming the protective effect of AKG on liver mitochondria in metabolically aberrant mice. However, as the last complex in the mitochondrial respiratory chain, we noted that the activity of complex V did not improve significantly after AKG pretreatment, which may be due to other effects of AKG on the subunits of complex V other than ATP5A. This warrants further investigation.

Notably, we confirmed that AKG promoted the activity of mitochondrial complex I, the main site of ROS production in mitochondria [31]. Increasing ROS has been proven to impede mitochondrial oxidative phosphorylation and TCA cycling, leading to decreased AKG levels *in vivo* [11,32]. Our results manifested that AKG pretreatment alleviated ROS accumulation in both liver and hepatocytes. We thereby hypothesized the addition of AKG could ameliorate the increase in mitochondrial superoxide due to lipid overload, which was confirmed by the mitoSOX staining in HepG2 cells. Increasing mitochondrial oxidative status aggravated the oxidation of mitochondrial protein [33]. As expected, the injection of P407 led to a dramatic increase in carbonylated proteins, as well as inhibited the activities and protein content of the mitochondria antioxidant enzymes Catalase and SOD, while these oxidative stress indexes could be effectively prevented by AKG pretreatment. We noticed that in the hyperlipidemic mouse liver, AKG mainly promoted the activity of mitochondria SOD, which is SOD2, but had no significant effect on SOD1. Not only that, although AKG supplementation promoted the upregulation of SOD2 expression, a much greater increase was observed in its activity, hinting at the possibility that SOD2 activity may receive regulation by post-translational modifications. Acetylation modification is important in the regulation of SOD2 activity [33]. We found that AKG inhibited SOD2 acetylation in the liver of hyperlipidemic mice, suggesting that deacetylation may be the primary cause of promoting SOD2 activation.

Nrf2 has been reported as a hinge in regulating the activity of antioxidant enzymes such as SOD. Our recent study reported that AKG mitigated endothelial oxidative stress and mitochondrial dysfunction in P407-induced hyperlipidemic mice via activated Nrf2 [11]. Here, we found that AKG restored lipid overload-induced loss of Nrf2 and its phase II enzyme proteins in either the liver or hepatocytes, as well as promoting Nrf2 entry into the nucleus, which further activated the expression of oxidative stress-related proteins. Meanwhile, considering that AKG suppressed the protein expression of Keap1 in the liver, it implies that AKG may be involved in regulating the ubiquitination of Nrf2. As predicted, AKG inhibited endogenous ubiquitination accumulation in lipid-overloaded hepatocytes, suggesting that AKG is likely to ameliorate fatty liver via deubiquitination of Nrf2.

It has been shown that AKG extends the lifespan of Drosophila by promoting AMPK phosphorylation [34]. AMPK activation plays a crucial role in the activation of PGC-1α and Nrf2 in mitochondrial protection [35]. We observed the activating effect of AKG on AMPK phosphorylation in dyslipidemia hepatocytes, which was evident in both *in vitro* and *in vivo* models. We therefore delve deeper into the involvement of AMPK activation in the regulatory role of AKG in liver protection and found that inhibited AMPK phosphorylation significantly compromised AKG's ability to reduce TG, T-CHO, and ROS levels in PA-injured HepG2 cells, also negated the upregulation of PGC-1α and Nrf2 and downregulation of ACC. Substantiated the involvement of AMPK activation in the process by which AKG ameliorates hepatic lipid metabolism abnormalities. Previous studies have highlighted that overexpression of both PGC-1α and Nrf2 has contributed to liver protection [36,37]. We proposed that mitochondrial and Nrf2-regulated oxidative stress may collaboratively form a mutually regulating circuit, representing an effective strategy for modulating the metabolic syndrome [38]. Since we observed that AKG simultaneously promotes the protein expressions of PGC-1α and Nrf2 in lipid-overloaded hepatocytes both *in vitro* and *in vivo*, we have tried to elucidate the respective roles of PGC-1α and Nrf2 in AKG's ability to ameliorate hepatocyte lipid metabolism abnormalities. After knockdown the PGC-1α and Nrf2 by siRNA, we found that depleted PGC-1α significantly blocked the inhibitory effect of AKG on PA-induced ROS and lipid accumulation, while silenced Nrf2 just prevented the inhibition of AKG on ROS. Simultaneous knockdown of PGC-1α and Nrf2 more significantly inhibited the ameliorative effect of AKG on lipid overload and oxidative stress. These findings suggest that AKG represents a protective effect on fatty liver through the AMPK-PGC-1α/Nrf2 signaling pathway.

In summary, our study establishes that AKG alleviates hyperlipidemia-induced fatty liver by modulating mitochondrial function and redox homeostasis mainly through the AMPK-PGC-1α/Nrf2 pathway. Given the fact that AKG is an endogenous mitochondrial nutrient characterized by lower toxicity and fewer side effects, our findings present compelling evidence supporting the potential therapeutic application of AKG in preventing NAFLD.

## 4 Materials and methods

### 4.1 Antibodies and reagents

AKG (≥98.5 %, catalog, K1128), P407 (catalog, 16758), and palmitic acid (PA, catalog, P9767) were purchased from Sigma (St. Louis, MO). Antibodies against PGC-1a (catalog, 66369-1) and Mouse IgG (B900620) was provided by Proteintech Group, Inc (Wuhan, China). Antibodies against Nrf2 (catalog, sc-365949), HO-1 (catalog, sc-390991), NQO1 (catalog, sc-376023), Mitofusin-1 (Mfn1, catalog, sc-166644), Mitofusin-2 (Mfn2, catalog, sc-100560), dynamin-related protein 1 (Drp1, catalog, sc-271583) and protein A/G PLUS-Agarose were purchased from Santa Cruz Biotechnology (Santa Cruz, CA). Antibodies against Mn-SOD (SOD2, catalog, ab137037), acetylate SOD2 (acetyl K68, catalog, ab137037), optic atrophy 1 (OPA1, catalog, ab157457) and Histone H3 (catalog, ab1791) from Abcam (Cambridge, United Kingdom). Antibodies against FASN (catalog, 3189), ACC (catalog, 3676), TFAM (catalog, 8076), Catalase (catalog, 14097) and Phospho-(Ser/Thr) Phe (catalog, 9631) was purchased from Cell Signaling Technology (Beverly, MA). Antibodies against complexes I (NDUFS3, 39 kDa, catalog, 459130), II (SDHB, 30 kDa, catalog, 459230), III (UQCRC1, 51 kDa, catalog, 459140), IV (MTCO1, 40 kDa, catalog, 459600), and V (ATP5A, 55 kDa, catalog, 459240), Lipofectamine RNAiMAX transfection reagent, MitoTracker Red and MitoSOX Red mitochondrial superoxide indicator was provided by Thermo Fisher Scientific (Carlsbad, CA). Assay kits for TG, T-CHO, FFA, MDA, CAT, ATP, and LPO were purchased from Jiancheng Biochemical, Inc. Ltd. (Nanjing, China). Nuclear and Cytoplasmic Protein Extraction Kits and Assay kits for total-SOD, SOD1, and SOD2 were obtained from Beyotime Biotechnology Co., Ltd. (Shanghai, China). AMPK-inhibitor Compound C was purchased from MedChemExpress (MCE, Monmouth Junction, NJ). Proteasome inhibitor MG132 was purchased from Selleck Chemicals (Houston, TX). Western and IP lysis buffer from Beyotime (Jiangsu, China). Prime-Script RT-PCR Kit from Takara (Otsu, Shiga, Japan).

### 4.2 Animal experiments

Male C57BL/6J mice (8 weeks) were purchased from Beijing Vital River Laboratory Animal Technology Co., Ltd. (Beijing, China) and randomly divided into four groups: control group, AKG group, P407 group, and P407+AKG group (n ≥ 6 in each group). According to our previous method [11], supplementation with 50 mg/kg AKG upregulated plasma AKG levels while inhibiting lipid overload. Here, the methodology of the previous animal experiments was replicated that for the first nine days, mice in the AKG group and the AKG + P407 group were gavaged 50 mg/kg/day AKG, while mice in the control group and the P407 group were gavaged with an equal volume of water. On the 10th day, the mice in the P407 group and P407+AKG groups were injected intraperitoneally with P407 0.5 g/kg for 24 h. The control group and AKG groups were injected with equal amounts of saline [11,39].

Blood samples were centrifuged at 5000 rpm for 15 min at 4 °C after orbital blood sampling, and the supernatant (serum) was gathered and stored at −80 °C for subsequent analysis as described in the instruction manual from Jiancheng Biochemical, Inc. Ltd. (Nanjing, China). The mice were sacrificed by cervical dislocation immediately after blood collection, and then liver tissue was separated and removed carefully. All operations were licensed by the Animal Care and Use Committee of Xi'an Jiaotong University (No. 2023–69). Efforts have been made in this study to minimize animal suffering and the number of animals used.

### 4.3 Cellular experiments

HepG2 cells were purchased from ATCC (Manassas, VA). Cells were cultured in Dulbecco's Modified Eagle's Medium (DMEM) supplemented with 10 % fetal bovine serum. Cells were cultured in a humidified atmosphere with 95 % air and 5 % CO_2._ When the HepG2 cell density reached approximately 40 %, the cells were pretreated with or without AKG for 24 h, followed by 250 μM PA challenged for 24 h. Compared with the PA-injured group, the control group added an equal amount of BSA [6,11].

Primary hepatocyte preparation: Male C57BL/6J mice (8-week-old) were anesthetized by chloral hydrate followed by extraction of primary hepatocyte according to the published method [40]. Briefly, the liver was perfused with Hanks’ Balanced Salt Solution (HBSS). After sufficient perfusion of the whole liver, type IV collagenase was used to digest. The digested liver tissue was filtered and centrifuged, and the resulting precipitate was the primary hepatocyte. Obtained primary hepatocytes were cultured on collagen-coated plates with a density of approximately 5 × 10^5^/ml. According to the references, this study was carried out with 100 μM AKG for 6 h followed by 250 μM PA treatment for 24 h after cell attachment [12].

### 4.4 Biochemical analysis

Livers isolated from mice were homogenized to centrifugation, then the supernatant was detected, collected serum was measured directly. TG, T-CHO, non-esterified fatty acid, AST, ALT, ATP, LPO, MDA, and catalase activity were measured by the commercially available kit from Nanjing Jiancheng Technology Co., Ltd. (Nanjing, China). Total-SOD, SOD1, and SOD2 activity was measured by the commercially available kit from Beyotime Biotechnology Co., Ltd. (Shanghai, China). Protein carbonyl was calculated using the commercially available kit from Beijing Solarbio Science & Technology Co., Ltd. (Beijing, China), and specific steps were followed according to the corresponding manufacturer's instructions.

### 4.5 Determination of ROS

Intracellular ROS and mitochondrial ROS were measured by the formation of fluorescent H_2_DCF-DA (2′,7′-Dichlorodihydrofluorescein diacetate) and MitoSOX, respectively. In a dark place, liver tissues or HepG2 cells were incubated with 10 μM DCFH2-DA or 5 μM of MitoSOX dissolved in the serum-free medium for 30 min. The fluorescence intensity was determined with a microplate fluorometer (Fluoroskan Ascent; Thermo Fisher Scientific, Inc.) at the wavelengths of 488 nm (excitation) to 535 nm (emission) for DCFH_2_-DA, at the wavelengths of 514 nm (excitation) to 580 nm (emission) for MitoSOX [41]. The ROS levels were quantified by the relative fluorescence per microgram of protein.

### 4.6 Liver histological analysis

The collected livers were placed in 4 % paraformaldehyde. Hematoxylin and Eosin (H&E) stained sections were prepared by Wuhan Servicebio Technology Co., Ltd. (Wuhan, China).

### 4.7 Western blot analysis

To obtain homogenization, liver tissue or HepG2 cell or liver primary cell samples were lysed with IP lysis buffer. The homogenates were centrifuged at 12,000 g for 15 min (4 °C). Supernatants were collected as whole-cell proteins. Samples of nuclear and cytoplasmic proteins were prepared according to the instructions using the Nuclear and Cytoplasmic Protein Extraction Kits provided by Beyotime Biotechnology Co., Ltd. (Shanghai, China). The BCA Protein Assay kit was used to determine the protein concentrations. Equally protein aliquots (10 μg) were separated by SDS-PAGE, then transferred to nitrocellulose membranes and blocked with 5 % nonfat milk dissolved in TBST buffer at room temperature for 1 h. Membranes were then incubated with primary antibodies at 4 °C overnight and then incubated with secondary antibodies at room temperature for 1 h. Antibodies were diluted with 1 % BSA in the ratio recommended in the instruction manuals. Chemiluminescent detection was visualized using an enhanced chemiluminescence reagent (Thermo Corporation, Rockford, IL) and quantified by scanning densitometry. The protein levels were quantified by the density detected by ImageJ software. The expression of each protein was adjusted to that of GAPDH as a loading control.

### 4.8 Mitochondrial complex activities

Liver mitochondria were isolated as previously described [33]. Briefly, liver tissue was homogenized by the hypotonic buffer (10 mmol/l NaCl, 2.5 mmol/l MgCl2, 10 mmol/l Tris-base, pH 7.5) on ice with a glass homogenizer (Fisher Scientific, Pittsburgh, PA). The homogenates were further centrifuged at different speeds to collect mitochondria for detection. The activities of complex I (NADH-ubiquinone reductase), complex II (succinate-ubiquinone oxidoreductase), complex III (ubiquinone-cytochrome c reductase), complex IV (cytochrome *c* oxidase), and complex V (ATP synthase) were determined according to methods previously reported [42].

### 4.9 Mitochondrial DNA (mtDNA) copy number

mtDNA copy number was determined by Real-time PCR. Total DNA was isolated by the QIAamp DNA Mini Kit (Qiagen, Hilden, Germany) and using mitochondrial D-loop primers as mtDNA copy numbers. The data were adjusted to 18s rRNA and further were analyzed by the 2-ΔΔCt method. The sequences of primers are presented in Supplementary Table S1.

### 4.10 Cell viability assay

Cell viability was determined by the MTT (3-[4,5-dimethylthiazol-2-yl]-2,5 diphenyl tetrazolium bromide) method. HepG2 cells were pretreated for 24 h with AKG in 96-well plates followed by 500 μM PA challenge for another 24 h, then 200 μL of MTT was diluted in DMEM (1:9) and added to each well. The plates were incubated for 4h. The absorbance was measured at a wavelength of 550 nm.

### 4.11 Mitochondria membrane potential (MMP)

The MMP was determined by lipophilic cationic probe JC-1 (5,5′,6,6′-tetrachloro-1,1′,3,3′-tetraethyl-benzimidazolyl-carbocyanine iodide). HepG2 cells were spread in 96-well plates. After treatment with AKG and PA, flushed off the medium, washed again with PBS, then stained with JC-1 dye for 1h. After JC-1 staining, the fluorescence wavelengths of 525 nm and 590 nm emission were detected under excitation luminescence of 488 nm. The result was presented by the calculated fluorescence value of 590 nm/525 nm.

### 4.12 Mito-tracker red staining

A mixture of Mito-tracker red dye (Invitrogen Inc., CA, USA) and serum-free H-DMEM was prepared at a ratio of 1:4000 for use. After AKG and PA treatments, HepG2 cells were washed once with sterile PBS, then 500 μL of the dye mixture was added to each well (12-well plate) under a light-avoiding environment, followed by 10 min incubation in the cell culture incubator. After staining, the dye mixture was discarded, and the cells were washed three times with PBS. After that, serum-free H-DMEM was added to each well. The stained cells were placed under a fluorescence microscope to observe the mitochondrial morphology. The mitochondrial network in the cell was accessed semi-automatically by the Mitochondrial Network Analysis (MiNA) toolset, a macro that utilizes the existing ImageJ plug-in to analyze the obtained Median Branch Length for quantitative analysis.

### 4.13 Small interfering RNA transfection

The small interfering RNA (siRNA) oligos were purchased from Genepharma Co. (Shanghai, China). The target sequences of PGC-1α siRNA were 5′-CCA CCA CUC CUC CUC AUA ATT-3’ (sense), 5′-UUA UGA GGA GGA GUG GUG GTT -3’ (antisense), and Nrf2 siRNA were 5′-CCA GAA CAC UCA GUG GAA UTT-3′(sense), 5′-AUU CCA CUG CUG AGU GUU CUG GTT-3’ (antisense). HepG2 cells were seeded in 6-well or 12-well plates and were transfected with a density of around 30 %. Lipofectamine RNAiMAX transfection reagent was mixed with siRNA to transfect into cells according to the manufacturer's instructions, and the final concentration of siRNA at the time of transfection was 50 nM. HepG2 cells were harvested or follow-treated with AKG and PA after being transfected for 48h.

### 4.14 Immunoprecipitation

Liver tissue or HepG2 cells were lysed with IP lysis buffer to make the homogenates and centrifuged at 12,000 g for 15 min (4 °C). The supernatant was collected as total protein and quantified by the BCA method. Protein concentration was uniformly adjusted to 2000 μg/mL. Add 20 μL Protein A + G Agarose with slow shaking at 4 °C for 2 h to remove nonspecific binding. The sample obtained after Pre-clearing was centrifuged at 2500 rpm at 4 °C for 5 min, then 20 μL of supernatant was taken to prepare the Input sample as a positive control group. To the remaining protein sample, 500 μg of them were collected and added with the antibody for the enrichment of purified proteins or IgG (1 μg), shaken slowly at 4 °C overnight. The next day, 20 μL of Protein A + G Agarose was added and slowly shaken for 2 h at 4 °C. Next, the precipitate was centrifuged at 2500 rpm for 5 min at 4 °C to collect the agarose bead-antigen-antibody complex. The final precipitate was used as the immunoprecipitated samples for Immunoblotting to detect post-translational modifications of the target protein.

### 4.15 Statistical analysis

This study used randomization and blinded analysis with an equal number of groups. One-way ANOVA, followed by Bonferroni's post hoc analysis was used for statistical analyses in all the experiments. *p* < 0.05 was considered statistically significant in all analyses. Data from at least three independent experiments are presented as the mean ± SEM.

## CRediT authorship contribution statement

**Danyu Cheng:** Conceptualization, Formal analysis, Investigation, Validation, Visualization, Writing – original draft. **Mo Zhang:** Investigation, Writing – review & editing. **Yezi Zheng:** Investigation. **Min Wang:** Investigation, Writing – review & editing. **Yilin Gao:** Investigation, Writing – review & editing. **Xudong Wang:** Investigation, Writing – review & editing. **Xuyun Liu:** Writing – review & editing. **Weiqiang Lv:** Investigation. **Xin Zeng:** Investigation. **Konstantin N. Belosludtsev:** Writing – review & editing. **Jiacan Su:** Funding acquisition, Writing – review & editing. **Lin Zhao:** Supervision, Writing – review & editing. **Jiankang Liu:** Conceptualization, Funding acquisition, Project administration, Resources, Supervision, Writing – review & editing.

## Declaration of competing interest

The authors declare that they have no known competing financial interests or personal relationships that could have appeared to influence the work reported in this paper.

## Abbreviations Used:

ACC: acetyl coenzyme A carboxylase
AKG: α-Ketoglutarate
AMPK: AMP-activated protein kinase
Drp1: Dynamin-related protein 1
ETC: mitochondrial electron transport chain
FFA: non-esterified fatty acids
HBSS: Hanks' Balanced Salt Solution
HMGCR: 3-hydroxy-3-methyl-glutaryl-CoA reductase
H&E: Hematoxylin and Eosin
Mfn1: Mitofusin-1
Mfn2: Mitofusin-2
mtROS: mitochondrial ROS
MMP: Mitochondria membrane potential
mtDNA: mitochondrial DNA
NAFLD: non-alcoholic fatty liver disease
Nrf2: NF-E2-related factor 2
PGC-1α: peroxisome proliferator-activated receptor γ-coactivator 1-α
P407: Poloxamer 407
ROS: reactive oxidative species
siRNA: small interfering RNA
SOD2: Mn-SOD
TCA: tricarboxylic acid
T-CHO: cholesterol
TG: triglyceride
