# Supplementary material for: α-Ketoglutarate prevents hyperlipidemia-induced fatty liver mitochondrial dysfunction and oxidative stress by activating the AMPK-pgc-1α/Nrf2 pathway
Source: Redox Biol. 2024 Jun 13;74:103230. doi: 10.1016/j.redox.2024.103230 (PMC11226981; doi:10.1016/j.redox.2024.103230)
Supplement: Multimedia component 1 [file mmc1.doc]

**Supplementary figures**

**
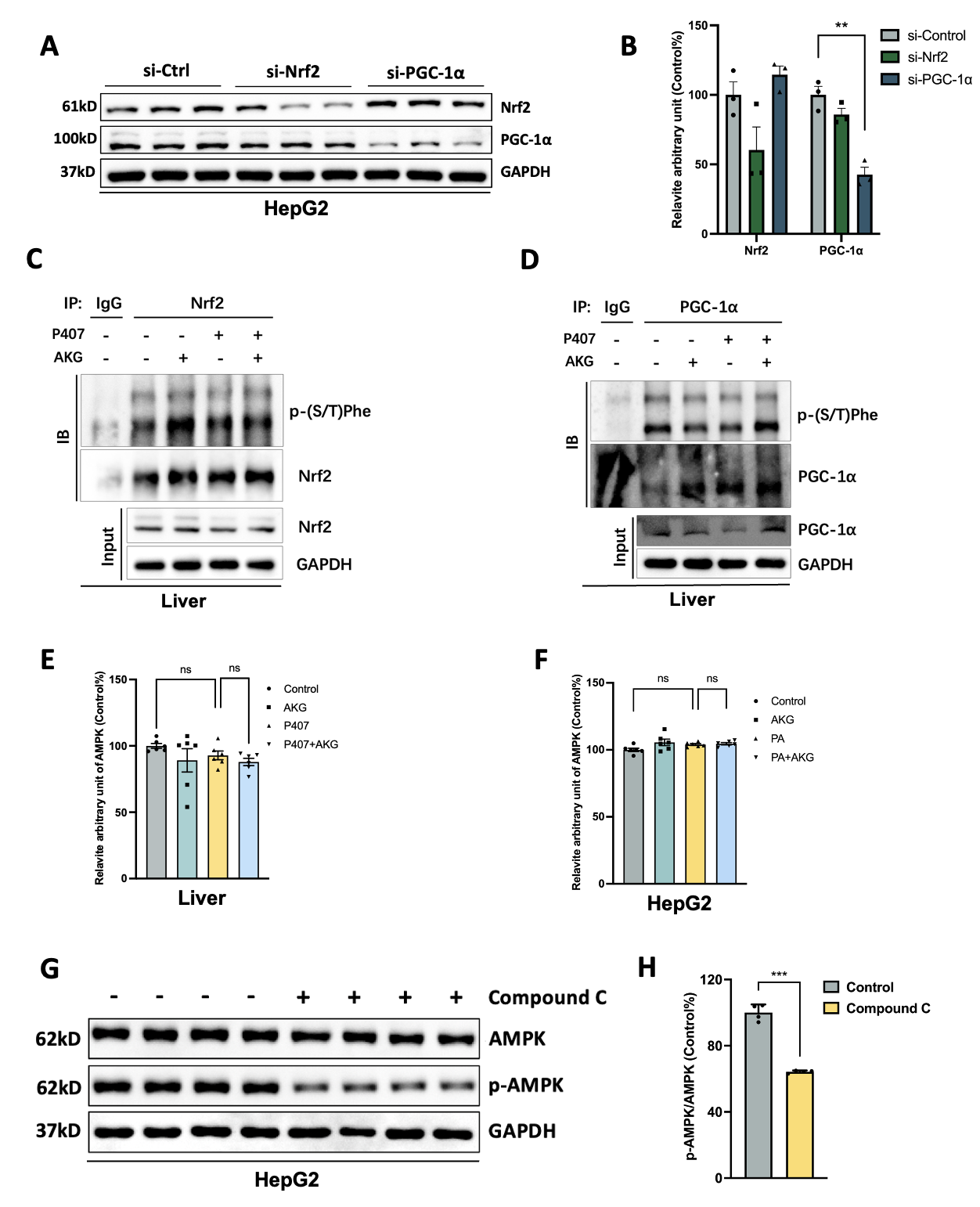
**

**Legend for Supplementary figures:** HepG2 cells were treated with the Ctrl siRNA, PGC-1α/Nrf2 siRNA for 48 h. (A) Western blot image and (B) Statistical analysis on relative protein levels of PGC-1α and Nrf2. (C) Immunoprecipitated Nrf2 in liver then detect the p-(S/T)phe of endogenous Nrf2 by Immunoblot analysis. (D) Immunoprecipitated PGC-1α in liver then detect the p-(S/T)phe of endogenous PGC-1α by Immunoblot analysis. Statistical analysis on the protein content of AMPK in (E) mouse liver and (F) HepG2 cell. HepG2 cells were treated with or without AMPK inhibitor Compound C (10 μM) for 1h. (G) Western blot image and (H) Statistical analysis on relative protein levels of p-AMPK/AMPK.

**Supplementary Table 1**

| Primer | Species | Primer（5’-3’） |
| --- | --- | --- |
| *D-loop* | Mouse | F: AGGCATGAAAGGACAGCA  R: TTGGCATTAAGAGGAGGG |
| *18s rRNA* | Mouse | F: GTAACCCGTTGAACCCCATT  R: CCATCCAATCGGTAGTAGCG |
| *D-loop* | Human | F: CAGTACCTAACAAACCCACA  R: GAGGTCGTAAACCCTATTGT |
| *18s rRNA* | Human | F: CAGCCACCCGAGATTGAGCA  R: TAGTAGCGACGGGCGGTGTG |
